# Supplementary material for: Multi-view data visualisation via manifold learning
Source: PeerJ Comput Sci. 2024 May 24;10:e1993. doi: 10.7717/peerj-cs.1993 (PMC11157621; doi:10.7717/peerj-cs.1993)
Supplement: Supplemental Information 1 [file peerj-cs-10-1993-s001.pdf]

1 ALGORITHMS

Data:M data sets, $X^{(m)} \in \mathbb{R}^{N \times p_m}, \quad \forall m \in \{1, \cdots, M\}$
Parameters:$Perp$ [Perplexity]; T [Number of iterations]; η [Learning rate]; $\alpha(t)$ [Momentum]
Result: Induced embedding, $Y \in \mathbb{R}^{n \times d}$. Often, $d = 2$
begin
    Optional step of implementing PCA, or multi-CCA on $X^{(m)} \quad \forall m \in \{1, \cdots, M\}$
    Compute pairwise affinities $p^m_{i|j}$ with perplexity $Perp, \quad \forall m \in \{1, \cdots, M\}$
    Set $p^m_{ij} = \frac{p^m_{i|j} + p^m_{j|i}}{2n}, \quad \forall m \in \{1, \cdots, M\}$
    Initialise solution $Y^{(0)} \sim \mathcal{N}(0, 0.1)$
    for *t=1 to T* **do**
        Compute induced affinities $q_{i|j}$ and set sum of gradients, $G = 0$
        for *m=1 to M* **do**
            Compute gradient $\frac{\delta C_m}{\delta Y}$
            $G \leftarrow G + \frac{\delta C_m}{\delta Y}$
        end
        Set $Y^{(t)} = Y^{(t-1)} + \eta G + \alpha(t)(Y^{(t-1)} - Y^{(t-2)})$
    end
end

Algorithm 1: Multi-SNE

Data: M data sets, $X^{(m)} \in \mathbb{R}^{N \times p_m}, \quad \forall m \in \{1, \cdots, M\}$
Parameters: k [Number of neighbours]
Result: Induced embedding, $\hat{Y} \in \mathbb{R}^{n \times d}$. Often, $d = 2$
begin
    for *m=1 to M* **do**
        Find k nearest neighbours of $X^{(m)}$.
        Compute W^m by minimising equation (7).
    end
    Let $\hat{W} = \sum_m \alpha^m W^m$, where $\sum_m \alpha^m = 1$.
    Compute the d-dimensional embeddings $\hat{Y}$ by minimising equation (8) under $\hat{W}$.
end

Algorithm 2: multi-LLE

Data: M data sets, $X^{(m)} \in \mathbb{R}^{N \times p_m}, \quad \forall m \in \{1, \cdots, M\}$
Parameters: k [Number of neighbours]
Result: Induced embedding, $\hat{Y} \in \mathbb{R}^{n \times d}$. Often, $d = 2$
begin
  for *m=1 to M* **do**
    Construct a $N \times N$ neighborouhood graph, $G_m \sim (V, E_m)$ with samples represented by nodes.
    The edge length between k nearest neighbours of each node is measured by Euclidean distance.
  end
  Measure the average edge length between all nodes.
  Combine all neighborouhood graphs into a single graph, $\tilde{G}$.
  In $D_G \in \mathbb{R}^{|V| \times |V|}$, the computed shortest path distances between nodes in $\tilde{G}$ are stored.
  Compute the d-dimensional embeddings Y by computing $y_i = \sqrt{\lambda_p} u_p^i$, where λ_p is the pth eigenvalue in decreasing order of the the matrix $\tau(D_G)$ and u_p^i the ith component of pth eigenvector. The operator, τ is defined by $\tau(D) = -\frac{HSH}{2}$, where S is the matrix of squared distances defined by $S_{ij} = D_{ij}^2$, and H is defined by $H_{ij} = \delta_{ij} - \frac{1}{N}$.
end

Algorithm 3: multi-ISOMAP

2 DATA CLUSTERING EVALUATION MEASURES

Let $\mathbf{X} = \{X_1, \cdots, X_r\}$ be the true classes of the data and $\mathbf{Y} = \{Y_1, \cdots, Y_s\}$ the clusterings found on N objects. In this study, we assume to know the number of clusters and thus set $r = s$. Let n_{ij} be the number of objects in X_i and Y_j. A contingency table is defined as shown in Table S1.

Table S1. A contingency table for data clustering. X_i refers to the ith class (truth) and Y_j refers to the jth cluster. n_{ij} are the number of samples found in class i and cluster j. In this study, $r = s$ was taken, as K-means with the true number of classes known was performed.

	Y_1	Y_2	$\cdots$	Y_s	$\sum_j^s Y_j$
X_1	n_{11}	n_{12}	$\cdots$	n_{1s}	$\sum_i^r n_{1i} = a_1$
X_2	n_{21}	n_{22}	$\cdots$	n_{2s}	a_2
$\vdots$	$\vdots$	$\ddots$	$\vdots$	$\vdots$	$\vdots$
X_r	n_{r1}	n_{r2}	$\cdots$	n_{rs}	a_r
	$\sum_i^r n_{i1} = b_1$	b_2	$\cdots$	b_s	$\mathbf{S} = \sum_i^r \sum_j^s n_{ij}$

The formulas of the four measures used to evaluate data clustering are given below, with the terms defined in Table S1.

Accuracy (ACC)

$$(Acc) = \frac{\sum_i \sum_j 1\{i = j\} n_{ij}}{\mathbf{S}} \tag{1}$$

Normalised Mutual Information (NMI)

$$(NMI) = \frac{2I(\mathbf{X}, \mathbf{Y})}{H(\mathbf{X}) + H(\mathbf{Y})} \tag{2}$$

where $I(\mathbf{X}, \mathbf{Y})$ is the mutual information between $\mathbf{X}$ and $\mathbf{Y}$, and $H(\mathbf{X})$ is the entropy of $\mathbf{X}$.

Rand Index (RI)

$$(RI) = \frac{\binom{N}{2} - \left[\frac{1}{2}\sum_i (\sum_j n_{ij})^2 + \sum_j (\sum_i n_{ij})^2 - \sum_i \sum_j n_{ij}^2\right]}{\binom{N}{2}} \tag{3}$$

$$= \frac{\alpha + \beta}{\binom{N}{2}} \tag{4}$$

where α refers to the number of elements that are in the same subset in X and in the same subset in Y, while β is the number of elements that are in different subsets in X and in different subsets in Y.

Adjusted Rand Index (ARI)

$$(ARI) = \frac{\sum_i \sum_j \binom{n_{ij}}{2} - \frac{\sum_i \binom{a_i}{2} \sum_j \binom{b_j}{2}}{\binom{n}{2}}}{\frac{1}{2}\left[\sum_i \binom{a_i}{2} + \sum_j \binom{b_j}{2}\right] - \frac{\sum_i \binom{a_i}{2} \sum_j \binom{b_j}{2}}{\binom{n}{2}}} \tag{5}$$

3 SINGLE-CELL DATA

In the multi-omics single-cell data analysis, we used the publicly available data set provided by 10x Genomics for human peripheral blood mononuclear cells (PBMC) [1]. This data set can be downloaded and installed via the `R` package `SeuratData`, by running the command `InstallData("pbmcMultiome")`. In their vignette [2], Hoffman et al. (2021) explored this data set to demonstrate how to jointly integrate and analyse such data.

In this data set, scRNA-seq and scATAC-seq profiles were simultaneously collected in the same cells by 10x Genomics. Data on 11909 single cells are available on 36601 genes and 108377 peaks in scRNA-seq and scATAC-seq, respectively. Cells with zero summed expression, along all genes were removed, leaving us with 10412 cells. Pre-processing was employed via the `Seurat` package, following the steps performed by Hoffman et al. (2021). Firstly, we log-normalised both data-views and then selected features for each individual data-view. In feature selection, we aim to identify a subset of features with high variability across cells (using the functions `FindVariableFeatures` and `FindTopFeatures`) (Stuart et al., 2019).

The multi-omics single-cell data set consists of 19 imbalanced clusters that correspond to their corresponding cell types; we assume the annotations provided by `Seurat` to be accurate (Figure S1). To evaluate the clustering performance of multi-SNE, we took a balanced subset of the data. Cell-type clusters with less than 200 cells were removed entirely and we combined cells with cell types under the same hierarchy. For example, *Intermediate B*, *Naive B* and *Memory B* were combined to create a single cluster, *B cells*. Similarly, *CD4 Naive*, *CD4 TCM* and *CD4 TEM* were combined as *CD4 cells*. After this process, we ended up with a subset of 9105 single cells separated in 6 cell-type clusters (*B cell*, *CD14 Mono*, *CD4*, *CD8 Naive*, *CD8 TEM* and *NK*).

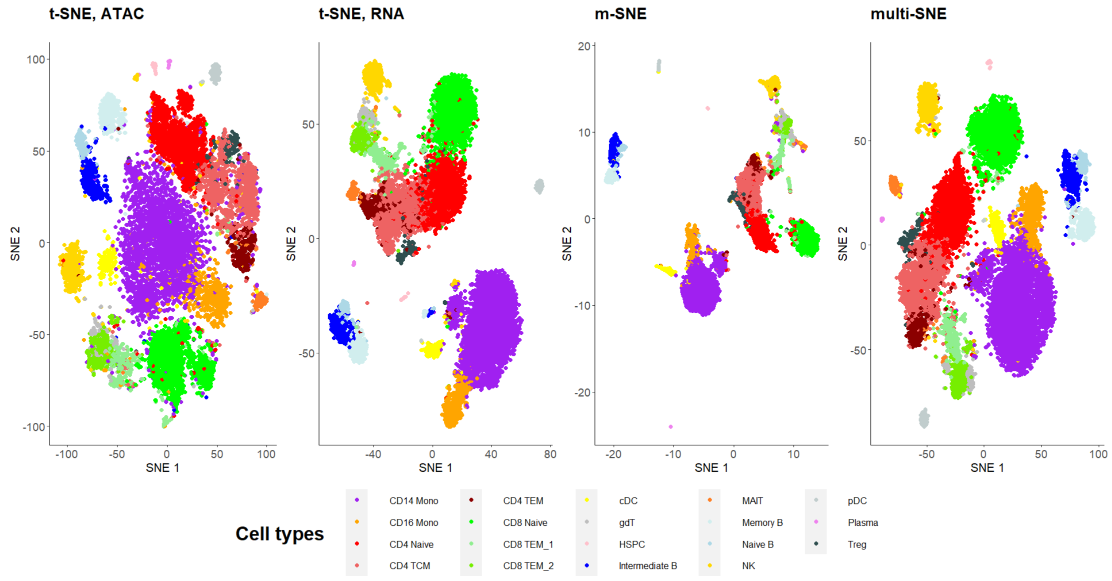

Figure S1. Visualisations of single-cell data. Projections of the full data set with unbalanced clusters produced by t-SNE on RNA, ATAC, m-SNE and multi-SNE on both data-views with perplexity $Perp = 80$ for the two t-SNE projections, $Perp = 100$ for m-SNE and $Perp = 20$ for multi-SNE.

M-SNE and multi-SNE combined the scRNA-seq and scATAC-seq to produce a more intelligible projection of the cells than t-SNE applied on either data-view. Qualitatively the superiority of the multi-view manifold learning algorithms may not be obvious at first, but subtle differences can be observed. Quantitatively, multi-SNE received the best evaluation scores, with $NMI = 0.807$, while m-SNE received $NMI = 0.760$. Single-view t-SNE scored $NMI = 0.620$ and $NMI = 0.572$ for scRNA-seq and scATAC-seq, respectively.

[1] `https://support.10xgenomics.com/single-cell-multiome-atac-gex/datasets/1.0.0/pbmc_granulocyte_sorted_10k`

[2] `https://satijalab.org/seurat/articles/atacseq_integration_vignette.html`

4 ADDITIONAL COMPARISONS

4.1 Multi-SNE, m-SNE and MV-tSNE2

This section justifies the exclusion of MV-tSNE2 from the comparisons against multi-SNE. Due to its superior performance, m-SNE was selected as an existing competitor of multi-SNE.

Multi-SNE and m-SNE outperformed MV-tSNE2 on all data sets presented in this manuscript (Figure S2). By comparing the produced visualisations on two data sets, Figure S2 evaluates the three algorithms qualitatively. Multi-SNE produced the best separation among clusters on both data sets. In MV-tSNE2, a lot of the samples are projected bundled together, making it difficult to distinguish the true clusters. Quantitative evaluation of the methods agree with the conclusions reached by assessing the visualisations qualitatively.

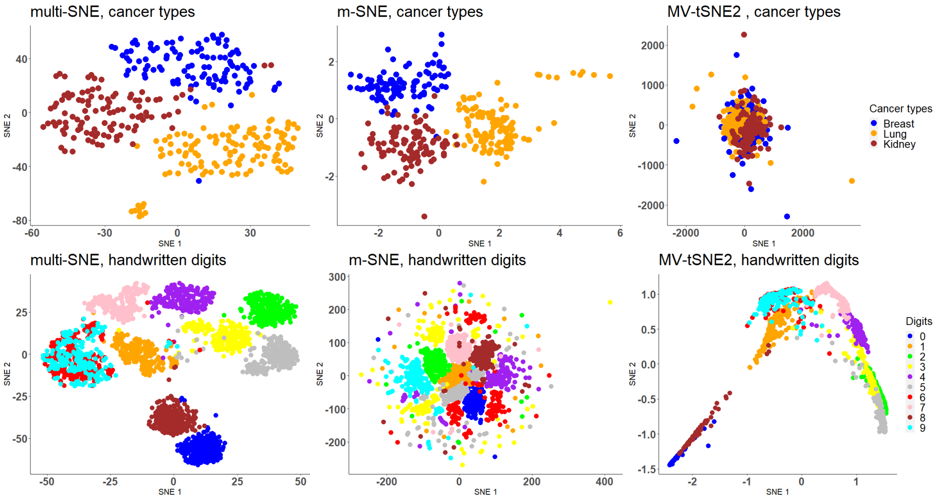

Figure S2. Visualisations by multi-SNE, m-SNE and MV-tSNE2. The three multi-view SNE-based projections of cancer types and handwritten digits data sets.

4.2 Multi-SNE, j-SNE and j-UMAP

At the same time as multi-SNE was developed, Canzar and Hoan Do (2021) proposed generalisations of t-SNE (named j-SNE) and UMAP (named j-UMAP) based on a similar objective function as multi-SNE. Canzar and Hoan Do (2021) introduced a regularisation term that reduces the bias towards specific data-views; the proposed objective function is given by:

$$C_{j-SNE} = \sum_m \sum_i \sum_j \alpha^m p_{ij}^m \log \frac{p_{ij}^m}{q_{ij}} + \lambda \sum_m \alpha^m \log \alpha^m, \quad (6)$$

where α^m represents the weight provided for the mth data-view and λ is a regularisation parameter. The weights and low-dimensional embeddings are updated iteratively. The adjustments on the weights of each data-view are performed in accordance to the regularisation parameter, which requires tuning for optimal results.

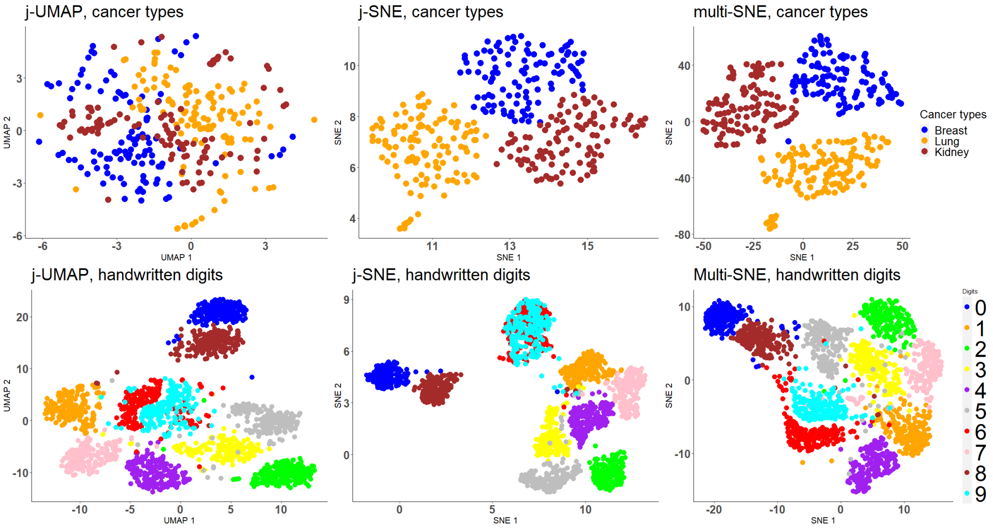

Figure S3. j-UMAP, j-SNE and multi-SNE visualisations. Projections of cancer types and handwritten digits data sets, produced by j-UMAP, j-SNE and multi-SNE.

Figure S3 compares qualitatively multi-SNE with j-SNE and j-UMAP (with their respective tuning parameters optimised) on the cancer types and handwritten digits data. As expected, the projections by j-SNE and multi-SNE are very much alike for both data sets. The increasing complexity imposed by the regularisation term in j-SNE does not seem to benefit the visualisation of the samples. j-UMAP does not separate the three cancer types, but it manages to separate the 10 digits, even samples that represent the 6 and 9 numerals; j-SNE failed to do that. This was achieved by multi-SNE at the 3-dimensional visualisation, or alternatively by using multi-CCA as a pre-training step. All three algorithms allocated similar weight values to each data-view on both data sets. In particular, transcriptomics on cancer types and morphological features on handwritten digits received the lowest weight.

4.3 Tuning parameters on real data

In this section, we have explored how the parameter values affect the multi-view approaches when analysing real data sets. Figure S4 depicts the NMI evaluation measure on each real data set for parameter values in the range S.

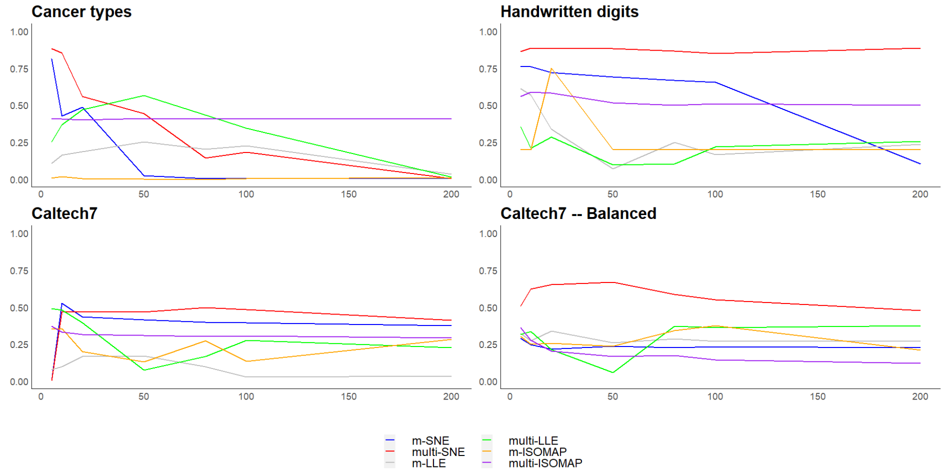

Figure S4. Real data sets evaluation via NMI. The NMI values are plotted against different parameter values on all multi-view manifold learning algorithms investigated in this manuscript.

Similar conclusions to the ones made in Section 4.3 were reached (Figure S4). SNE-based solutions had a more consistent performance than LLE and ISOMAP based approaches. In contrast to the conclusions reached by testing the tuning parameters on synthetic data, SNE-based approaches applied to the cancer types data set, performed the best when the perplexity was low. This observation highlights the importance of the tuning parameters (perplexity and the number of neighbours) in these algorithms, as discussed by their respective authors. For the remaining data sets, its performance was stable on different parameter values. With the exception of cancer types data, the performance of LLE-based solutions form similar behaviour with the synthetic data (*i.e.* their performance is reduced around $NN = 50$ and then it is regained.

4.4 Increased randomness in data

We have further explored how additional noise affects the performance of the multi-view learning approaches.

As discussed, the NDS data set contains three informative data-views and one noisy data-view. In Section 4.4, we concluded that the inclusion of the noisy data-view reduces the performance both qualitatively and quantitatively. This complication was targeted and solved through an automatic weight-updating approach in Section 4.5.1. The purpose of this section is to test the performance of multi-view manifold learning solutions on data sets with higher levels of randomness. To increase the noise in the synthetic data, additional noisy data-views were generated. In particular, this section compares the performance of manifold learning algorithms on three synthetic data sets: (a) NDS, (b) NDS with one additional noisy data-view, and (c) NDS with two additional noisy data-views. Each simulation was performed for 200 runs and with equal weights for a fair comparison.

Multi-SNE was the superior algorithm in all simulations (Table S2). With each additional noisy data-view, all multi-view manifold learning algorithms saw a reduction in their performance. Although all evaluation measures reflect this observation, the change in their performance is best observed on the NMI values (Table S2). Further, with more noisy data-views, the variance of the evaluation measures increased. This observation suggests that all algorithms clustered the samples with higher uncertainty.

The proposed automatic weight-adjusting process ensures that all data-views receive a weight value, which suggests that noisy data-views do not receive zero weight. For example, in the NDS with two additional noisy data-views scenario, this process returned higher weights for the informative data-views ($X1$, $X2$, $X3$), than the noisy ones ($X4$, $X5$, $X6$) (Figure S5). Although the weights between informative and noisy data-views are close in value, the proposed automatic weight-adjusting process can successfully distinguish informative from noisy data-views (Figures S5). To further assess if this process allocates substantial weight to noisy data-views, a scenario in which the data set contains more noisy data-views than informative ones was investigated.

In particular, a simulation was performed in which 1 data-view is informative and 2 are noisy. The informative data-view contains information to split the samples into 3 clusters, while the 2 noisy data-views assign all samples on the same cluster. Multi-SNE separates the samples by their respective cluster, despite having more noisy data-views than informative ones (Figure S6). In accordance with the other simulations, multi-SNE assigns a higher weight value on the informative data-view than on the noisy data-views. The informative data-view received a weight of 0.4 and each of the two noisy data-view 0.3 (Figure S6). This difference in the weights between the data-views acts as an incentive for the user to investigate the implementation of the algorithm by excluding the data-view(s) that received the lowest weight(s).

Table S2. Clustering performance of NDS and with additional noisy data-views. For each data set, red highlights the method with the best performance on each measure between each group of algorithms (SNE, LLE or ISOMAP based). The overall superior method for each data set is depicted with **bold**. The parameters *Perp* and *NN* refer to the selected perplexity and number of nearest neighbours, respectively. They were optimised for the corresponding methods.

Data Set	Algorithm	Accuracy	NMI	RI	ARI
NDS	SNE_{concat} [Perp=80]	0.747	0.628	0.817	0.598
	m-SNE [Perp=50]	0.650	0.748	0.766	0.629
	**multi-SNE** [Perp=80]	0.989	0.951	0.969	0.987
	LLE_{concat} [NN=5]	0.606	0.477	0.684	0.446
	m-LLE [NN=20]	0.685	0.555	0.768	0.528
	multi-LLE [NN=20]	0.937	0.768	0.922	0.823
	$ISOMAP_{concat}$ [NN=100]	0.649	0.528	0.750	0.475
	m-ISOMAP [NN=5]	0.610	0.453	0.760	0.386
	multi-ISOMAP [NN=300]	0.778	0.788	0.867	0.730
Higher dimension	SNE_{concat} [Perp=80]	0.723	0.648	0.787	0.585
	m-SNE [Perp=50]	0.623	0.705	0.734	0.605
	**multi-SNE** [Perp=80]	0.983	0.937	0.951	0.966
	LLE_{concat} [NN=5]	0.575	0.427	0.628	0.402
	m-LLE [NN=20]	0.671	0.534	0.755	0.513
	multi-LLE [NN=20]	0.903	0.788	0.898	0.802
	$ISOMAP_{concat}$ [NN=100]	0.622	0.510	0.705	0.453
	m-ISOMAP [NN=5]	0.589	0.439	0.734	0.344
	multi-ISOMAP [NN=300]	0.765	0.767	0.859	0.711
One additional noisy	SNE_{concat} [Perp=10]	0.650	0.522	0.724	0.489
	m-SNE [Perp=100]	0.689	0.584	0.786	0.530
	**multi-SNE** [Perp=50]	0.965	0.854	0.956	0.901
	LLE_{concat} [NN=10]	0.604	0.445	0.723	0.413
	m-LLE [NN=10]	0.667	0.522	0.765	0.490
	multi-LLE [NN=5]	0.912	0.692	0.891	0.756
	$ISOMAP_{concat}$ [NN=20]	0.543	0.375	0.733	0.481
	m-ISOMAP [NN=20]	0.552	0.482	0.703	0.444
	multi-ISOMAP [NN=5]	0.584	0.501	0.739	0.493
Two additional noisy	SNE_{concat} [Perp=10]	0.581	0.310	0.688	0.309
	m-SNE [Perp=10]	0.603	0.388	0.712	0.359
	**multi-SNE** [Perp=10]	0.936	0.781	0.926	0.832
	LLE_{concat} [NN=10]	0.523	0.251	0.641	0.222
	m-LLE [NN=10]	0.570	0.344	0.682	0.317
	multi-LLE [NN=5]	0.858	0.557	0.832	0.622
	$ISOMAP_{concat}$ [NN=20]	0.470	0.389	0.565	0.409
	m-ISOMAP [NN=20]	0.489	0.406	0.611	0.453
	multi-ISOMAP [NN=5]	0.524	0.467	0.782	0.517

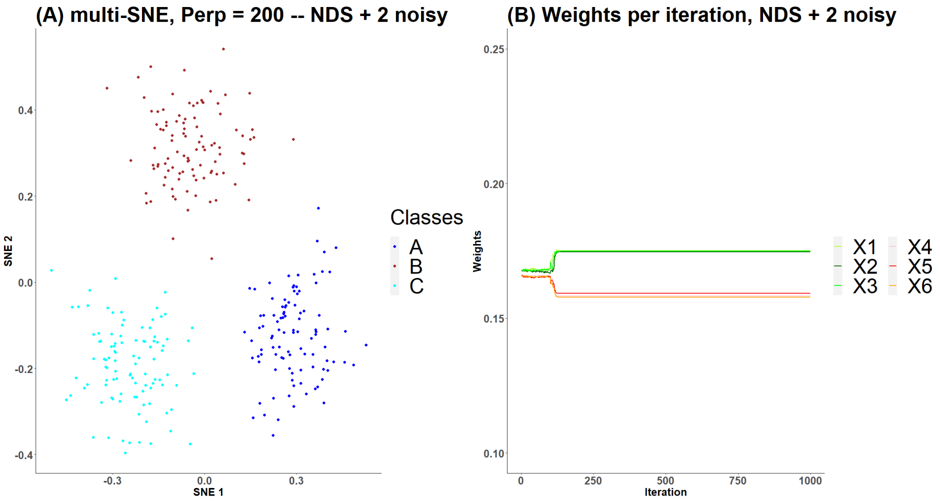

Figure S5. Visualisations on NDS with 2 additional noisy data-views. (A) Scatter-plot of the simulated samples obtained by multi-SNE with perplexity $Perp = 200$ and (B) Weights received by the algorithm on the 6 data-views. The first 4 follow the structure of NDS simulation; three informative data-views and a noisy one. Each informative data-view separates the samples differently, but taken together they are split equally into three clusters. $X5$ and $X6$ represent the 2 additional noisy data-views.

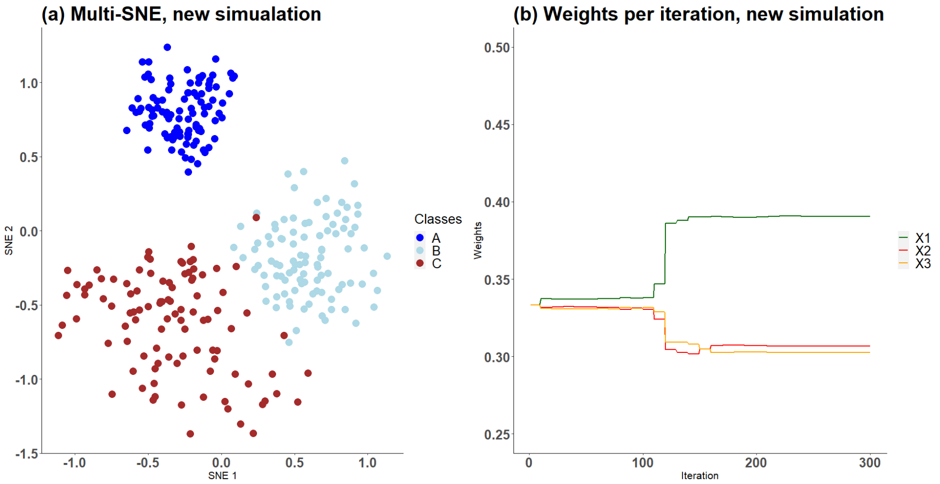

Figure S6. Visualisations on new simulation with 1 informative and 2 noisy data-views. (A) Scatter-plot of the simulated samples obtained by multi-SNE with perplexity $Perp = 100$ and (B) Weights received by the algorithm on the 3 data-views. The informative data-view contains information to split the samples into 3 clusters, while the 2 noisy data-views assign all samples to lie on the same cluster.

4.5 t-SNE on single-view cancer types

Table S3 presents the clustering performance of t-SNE applied on the three views in the cancer types data set, separately. Genomics was the favoured view on all evaluation measures.

Table S3. Clustering performance on the induced embedding of a single view obtained by implementing t-SNE on Cancer Types data. Standard deviation is reported in parentheses.

	ACC	NMI	RI	ARI
Genomics	0.595 (0.044)	0.299 (0.041)	0.667 (0.017)	0.253 (0.039)
Epigenomics	0.500 (0.036)	0.116 (0.033)	0.598 (0.018)	0.107 (0.035)
Transcriptomics	0.456 (0.023)	0.042 (0.011)	0.572 (0.006)	0.049 (0.013)

4.6 Handwritten digits projection in 3 dimensions (3D)

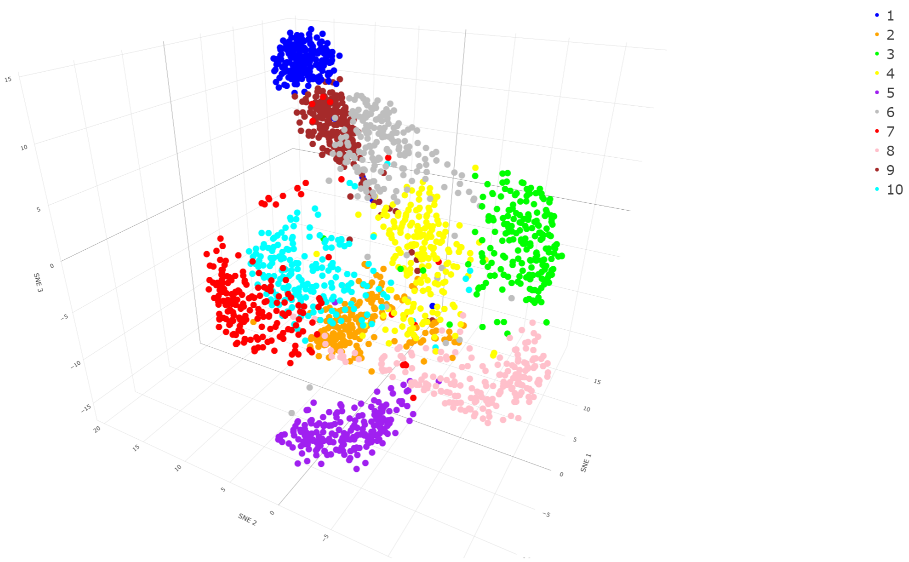

Figure S7. 3D multi-SNE visualisation of handwritten digits. Projections produced by weight adjusting multi-SNE with multi-CCA as pre-training and perplexity $Perp = 80$. Colours present the true clustering of the data points.

4.7 Alternative quantitative evaluation measures for clustering

Accuracy (ACC), Normalised Mutual Information (NMI), Rand Index (RI) and Adjusted Rand Index (ARI) are the evaluation measures chosen to quantitatively evaluate the clustering performance of the proposed multi-view approaches. These measures were chosen because the true annotations of the data sets are known and together they provide a wide assessment range. Practically, clustering is often applied to data with unknown annotations (labelling), therefore for completeness, we have further explored the implementation of the Silhouette score for identifying the optimal tuning parameter of the manifold visualisation approaches. The Silhouette score is a widely used measure for quantifying the clustering produced by the clustering algorithms, or for selecting the optimal number of clusters.

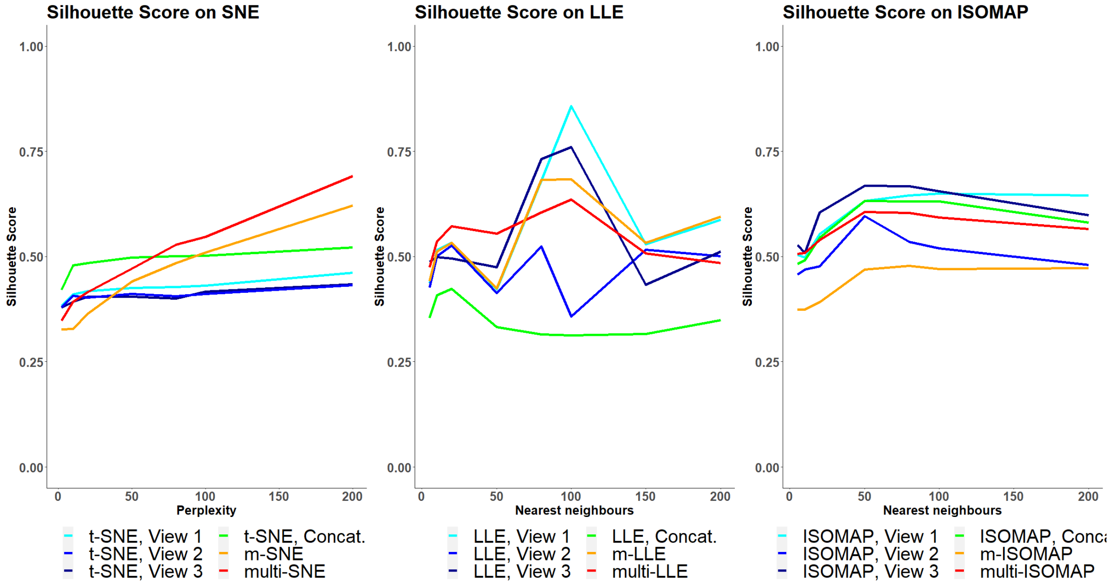

Figure S8. Silhouette score on MCS. The clustering evaluation via Silhouette score is plotted against different parameter values on all SNE, LLE and ISOMAP based algorithms.

Figure S8 presents the evaluation performance of the methods with respect to their tuning parameter when the Silhouette score is evaluated instead of the other four measures. This figure complements Figure 7. The Silhouette score is not always in agreement with the other evaluation measures. For example, in SNE-based solutions, according to the Silhouette score, multi-SNE is favoured over the other methods only when perplexity is 100. Another difference between the silhouette score and other measures is that as the perplexity increases, multi-SNE remains stable for the other measures (for $Perp \geq 50$), while its silhouette score keeps increasing. Silhouette score measures how well the clusters are separated between them. This is conceptually different from what the other measures quantify which is how well the proposed clusters agree with the known clusters. It is therefore of no surprise that the findings are not always in agreement.

4.8 Alternative clustering algorithms

For the clustering task of the samples, any clustering algorithm could have potentially been applied to the low-dimensional embeddings produced by the multi-view visualisation approaches proposed.

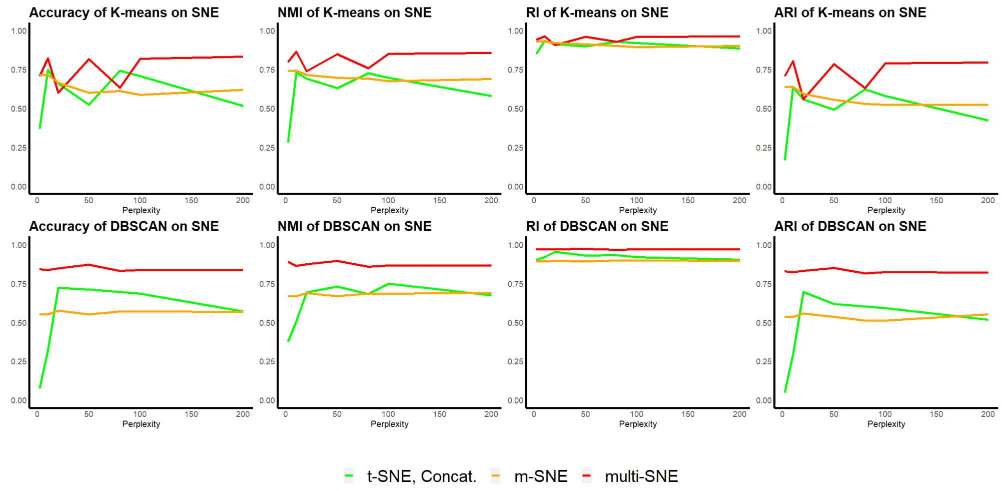

Figure S9. ***K*-means and DBSCAN on SNE-based solutions applied on handwritten digits data set.** The clustering evaluation measures are plotted against different perplexity values on multi-SNE, m-SNE and SNE_{concat}. The performance of K-means and DBSCAN applied on the produced embeddings is depicted in the first and second row of this figure, respectively

In the main body of this manuscript, the K-means algorithm was chosen due to its popularity, its strong robust performance and because the true number of clusters is known for all data sets. In practice, the latter is not always true, and clustering algorithms that do not require the number of clusters as a parameter input are preferable. Density-based spatial clustering of applications with noise (DBSCAN) is an example of such an algorithm (Ester et al., 1996). DBSCAN instead requires two other tuning parameters: the minimum number of samples required to form a dense cluster and a threshold in determining the neighbourhood of a sample, named ε.

The implementation of DBSCAN on handwritten digits, smooths the performance of SNE-based solutions across different perplexity values (Figure S9). For all parameter values, DBSCAN performs equally well, while the performance of K-means slightly oscillates.

A greater disagreement between the two unsupervised learning algorithms is observed in their application to caltech7 data set (Figure S10). While the accuracy of multi-SNE by implementing K-means reduces with higher perplexity, the opposite behaviour is observed when DBSCAN is implemented. In addition, DBSCAN finds multi-SNE to be superior over m-SNE, while K-means concludes the opposite.

This section demonstrates that the implementation of clustering on the produced embeddings is not restricted only to K-means, but alternative clustering solutions may be used. In particular, DBSCAN is a good choice, especially when the true number of clusters is unknown.

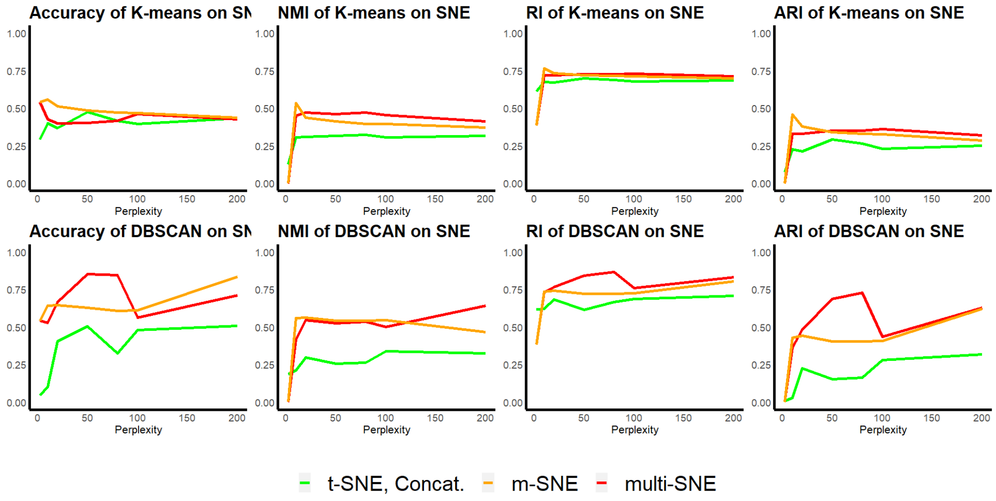

Figure S10. ***K*-means and DBSCAN on SNE-based solutions applied on caltech7 data set.** The clustering evaluation measures are plotted against different perplexity values on multi-SNE, m-SNE and SNE_{concat}. The performance of K-means and DBSCAN applied on the produced embeddings is depicted in the first and second row of this figure, respectively

5 REPRODUCIBILITY

The code of multi-SNE was based on the publicly available software, written by the author of t-SNE, found in the following link:
https://lvdmaaten.github.io/tsne/

In this manuscript, all t-SNE results were obtained by running the original R implementation (https://cran.r-project.org/web/packages/tsne/) and verified by the original Python implementation (https://lvdmaaten.github.io/tsne/).

We refer the readers to follow the code and functions provided in the link below to reproduce the findings of this paper. The software for m-SNE and m-LLE were not found publicly available and thus we used our own implementation of the method that can be found in the same link below:
https://github.com/theorod93/multiView_manifoldLearning

An R package that contains the code for multi-SNE can be installed via devtools and it can be found in https://github.com/theorod93/multiSNE

We refer the readers to follow the links provided in the main body of the paper for the public multi-view data used in this paper.

6 COMPUTATIONAL TIME AND SCALABILITY

In terms of computation time, none of the multi-view manifold learning algorithms was consistently faster than the rest (Table S4). However, multi-SNE was often the slowest algorithm, while m-SNE and multi-ISOMAP had the fastest computation time. The SNE-based solutions are based on the original t-SNE algorithm, as described in Section 5 of the Supplemental material.

Table S4. Averaged running time recorded in minutes. Taken for each manifold learning algorithm on all data sets seen in this paper; standard deviation is given in parentheses. All algorithms ran on High Performance Computing with 4 nodes.

**Running time**	MMDS	NDS	MCS	Caltech7	Handwritten Digits	Cancer Types
m-SNE	0.43 (0.019)	0.29 (0.07)	0.42 (0.01)	4.29 (0.54)	13.34 (3.43)	251.71 (15.68)
multi-SNE	1.07 (0.100)	0.78 (0.14)	1.02 (0.01)	15.95 (0.71)	45.76 (8.44)	252.00 (11.23)
m-LLE	0.25 (0.071)	0.40 (0.12)	0.42 (0.34)	37.5 (2.21)	26.28 (8.82)	159.52 (17.49)
multi-LLE	0.28 (0.099)	0.41 (0.15)	0.30 (0.14)	37.9 (2.57)	27.94 (5.29)	157.73 (18.19)
m-ISOMAP	0.22 (0.015)	0.57 (0.06)	0.37 (0.01)	38.07 (3.09)	29.52 (5.37)	154.83 (18.04)
multi-ISOMAP	0.24 (0.032)	0.54 (0.13)	0.33 (0.05)	21.23 (2.24)	16.77 (4.65)	85.47 (14.57)

We have further explored the scalability of the methods, and most specifically the effect of the sample size to the running time of the methods. For the purposes of this experiment, the Caltech7 dataset was utilised, where the running time of the methods was evaluated on different subsets of the dataset.

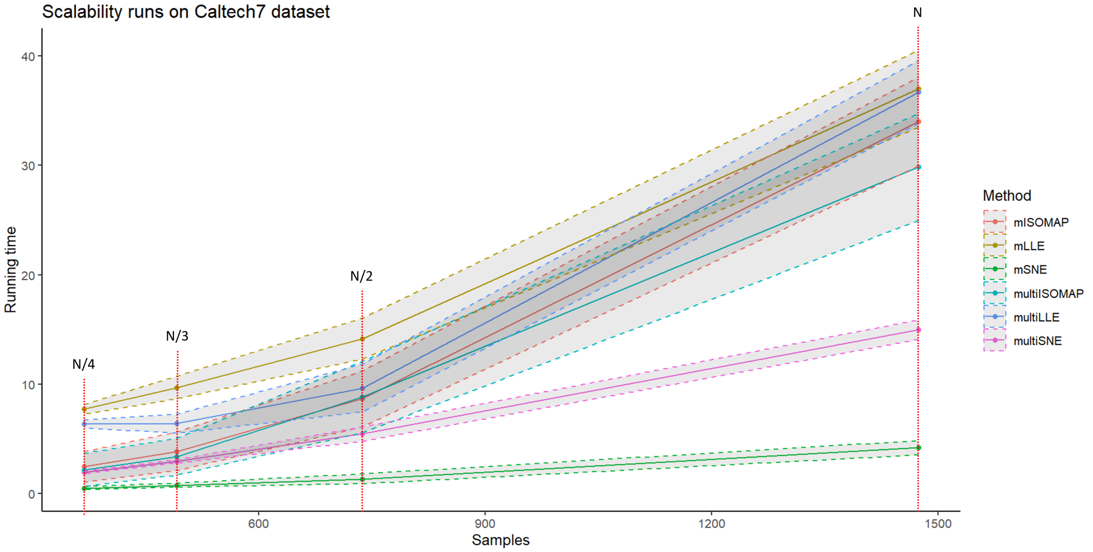

Figure S11. Scalability. Averaged running time recorded in minutes taken for each manifold learning algorithm on Caltech7 dataset with four different sets of samples: (i) All of the samples ($N = 1474$), (ii) Half of the samples ($N/2 = 737$), (iii) Third of the samples ($N/3 = 491$), and (iv) Quarter of the samples ($N/4 = 368$). All algorithms ran on High Performance Computing with 4 nodes. The shaded regions correspond to the standard deviation of the running time.

From Figure S11 it can be seen that the fastest algorithm is m-SNE that almost has a constant running time across the different sample sizes. The second fastest is the multi-SNE approach, on the other hand, the slowest running time is recorded by the m-LLE approach. The running time of the methods seems to follow a power law relationship with the number of samples, where the methods with lower exponents are more scalable with N. M-SNE is found to be the most scalable (0.77 power law exponent), followed by multi-SNE (1.96), while m-LLE had the largest exponent of 7.73. The power law exponents were computed by using the `R` package, `poweRlaw` (Gillespie, 2015).

REFERENCES

Canzar, S. and Hoan Do, V. (2021). A generalization of t-sne and umap to single-cell multimodal omics. *Genome Biology*, 22(1):1–9.

Ester, M., Kriegel, H.-P., Sander, J., and Xu, X. (1996). A density-based algorithm for discovering clusters in large spatial databases with noise. *Knowledge Discovery and Data Mining*, 34:226–331.

Gillespie, C. S. (2015). Fitting heavy tailed distributions: The poweRlaw package. *Journal of Statistical Software*, 64(2):1–16.

Hoffman, P., Lab, S., and Collaborators. (2021). Integrating scrna-seq and scatac-seq data. `https://satijalab.org/seurat/articles/atacseq_integration_vignette.html`. Accessed: 2021-03-18.

Stuart, T., Butler, A., Hoffman, P., Hafemeister, C., Papalexi, E., Mauck, W. M. r., Hao, Y., Stoeckius, M., Smibert, P., and Satija, R. (2019). Comprehensive integration of single-cell data. *Cell*, 177:1888–1902.
